# Enhanced Risk Stratification of Smoldering Multiple Myeloma with Dynamic Biomarkers: A Multinational, Multicenter Study including 2,270 Participants (PANGEA 2.0)

**DOI:** 10.21203/rs.3.rs-6696313/v1

**Published:** 2025-06-03

**Authors:** Floris Chabrun, Daniel Schwartz, Susanna Gentile, Elias Mai, Tulika Gupta, Jacqueline Perry, David Cordas Dos Santos, Thomas Hielscher, Annika Werly, Sophia Schmidt, Foteini Theodorakakou, Despina Fotiou, Christine Liacos, Nikolaos Kanellias, Noelia Gisbert, Esperanza Martin-Sanchez, Rosalinda Termini, Johannes Waldschmidt, Selina Chavda, Louise Ainley, Matteo Claudio Da Vià, Claudio de Magistris, Loredana Pettine, Michael Timonian, Jean-Baptiste Alberge, Vidhi Patel, Patrick Costello, Catherine Tobia, Sally Phan, Jennifer Lamb, Maria-Theresa Silverio, Maya Davis, Elizabeth O'Donnell, Catherine Marinac, Omar Nadeem, Niccolo Bolli, Kwee Yong, Martin Kortüm, Hermann Einsele, Maria Victoria Mateos Manteca, Shaji Kumar, Jesus San Miguel, Bruno Paiva, Efstathis Kastritis, Meletios Dimopoulos, Marc Raab, Lorenzo Trippa, Irene Ghobrial

**Affiliations:** Angers University Hospital; Dana-Farber Cancer Institute; Dana-Farber Cancer Institute; Heidelberg University Hospital; Dana Farber Cancer Institute; Dana-Farber Cancer Institute; Dana-Farber Cancer Institute; German Cancer Research Center (DKFZ); Heidelberg University Hospital and Medical Faculty Heidelberg; Heidelberg University Hospital and Medical Faculty Heidelberg; National and Kapodistrian University of Athens; National and Kapodistrian University of Athens; National and Kapodistrian University of Athens; National and Kapodistrian University of Athens; Cancer Center Clinica Universidad de Navarra; Cancer Center Clinica Universidad de Navarra; Cancer Center Clinica Universidad de Navarra; University Hospital Wuerzburg; University College London; University College London; Fondazione IRCCS Ca’ Granda Ospedale Maggiore Policlinico; Fondazione IRCCS Ca’ Granda Ospedale Maggiore Policlinico; Fondazione IRCCS Ca’ Granda Ospedale Maggiore Policlinico; Dana-Farber Cancer Institute, Harvard Medical School, Broad Institute of MIT & Harvard; Dana-Farber Cancer Institute; Dana-Farber Cancer Institute; Dana-Farber Cancer Institute; Dana-Farber Cancer Institute; Dana-Farber Cancer Institute; Dana-Farber Cancer Institute; Dana-Farber Cancer Institute; Dana-Farber Cancer Institute; Dana-Farber Cancer Institute; Dana-Farber Cancer Institute; Dana-Farber Cancer Institute; Department of Oncology and Hemato-Oncology, University of Milan, 20122 Milan, Italy.; University College London; University Hospital Würzburg; University Hospital Würzburg; Hospital Universitario de Salamanca, Instituto de Investigación Biomédica de Salamanca (IBASL), Centro de Investigación del Cáncer (IBMCC-USAL,CSIC); Mayo clinic; University of Navarra; Cancer Center Clinica Universidad de Navarra; National and Kapodistrian University of Athens School of Medicine; School of Medicine, National and Kapodistrian University of Athens; Heidelberg University Hospital, German Cancer Research Center (DKFZ); Dana-Farber Cancer Institute; Dana-Farber Cancer Institute

**Keywords:** Smoldering multiple myeloma, multiple myeloma, risk prediction, dynamic risk stratification

## Abstract

Accurate prediction of risk of progression from smoldering (SMM) to active multiple myeloma (MM) is paramount to individualized early therapeutic strategies with minimum risk of overtreatment. Current risk stratification models do not account for evolving biomarker trajectories. We assembled the largest cohort to date of 2,270 SMM patients from six international centers with longitudinal clinical and biological data to train and validate the PANGEA 2.0 risk models. Four evolving biomarkers were significantly associated with shorter time-to-progression: M-protein increase ≥0.2g/dL, involved:uninvolved serum free light chain ratio increase ≥20, creatinine increase >25%, and hemoglobin decrease ≥1.5g/dL. PANGEA 2.0 outperforms established models including the 20/2/20 and IMWG models by more accurately predicting progression (C-statistics=0.69–0.84), even without biomarker history (C-statistics=0.69–0.83) or recent bone marrow biopsy. PANGEA 2.0 is an easy-to-use, open-access tool (https://ghobrial.shinyapps.io/pangea_2_calculator) to improve and individualize SMM risk stratification. Validation tools are available to compare PANGEA 2.0 to established models (https://ghobrial.shinyapps.io/pangea_validation).

## Introduction

Smoldering multiple myeloma (SMM) is a biologically heterogeneous precursor to symptomatic multiple myeloma (MM), characterized by highly varying time to progression^1^. It is currently defined by the presence of a non-IgM M-protein ≥ 3 g/dL or urinary M-protein ≥ 500 mg/24 h and/or 10–60% clonal bone marrow plasma cells (BMPCs) without evidence of any myeloma-defining events^1^. Current models to stratify patients by the risk of progression from SMM to MM rely on static biomarkers, without using data on the preceding biomarker trajectory. The commonly used 20/2/20 model includes serum M-protein concentration (> 2 g/dL), involved over uninvolved (involved:uninvolved) serum free light chain (sFLC) ratio (> 20), and BMPC infiltration (> 20%), which are routinely measured in patients followed for SMM. The International Myeloma Working Group (IMWG) model additionally includes cytogenetic abnormalities such as t(4;14), t(14;16), del(13/13q), and gain(1q)^2, 3^.

These current risk prediction models are well established and provide simple, reliable clinical scores. However, they have several limitations. Most notably, they fail to account for evolving biomarkers that reflect dynamic disease progression^4-12^, and they require bone marrow biopsies, which are often unavailable during follow-up and may have inconsistent results due to the patchy nature of plasma cell infiltration^13^. As a result, some high-risk patients go undetected and develop end-organ damage such as bone lesions, despite close monitoring^14,15^. Conversely, other patients may be mistakenly labeled as high-risk due to the failure to account for stable biomarker trajectories that imply stable tumor mass over time^16^. Therefore, there is an urgent need for more accurate models that use longitudinal biomarker trajectories to identify SMM patients at highest risk of progression to overt MM, while minimizing the risk of overtreatment or underestimation of progression^17-21^. This is especially pressing in light of recent data showing the clinical benefit of early treatment with daratumumab in high-risk SMM.

To address these limitations, we analyzed a large cohort of 2,270 patients with untreated SMM to identify new predictive features from evolving biomarkers (including M-protein, sFLC ratio, creatinine, and hemoglobin) and assess their contribution to longitudinal risk assessment of progression from SMM to MM. The training cohort consisted of 1,031 patients at Dana-Farber Cancer Institute (DFCI), and the validation cohort comprised 1,239 patients from five international institutions. Our aim was to develop the PANGEA 2.0 model for repeated risk monitoring with significantly improved prediction accuracy compared to current models.

## Results

We first aimed to develop a risk stratification model using clinical and biological variables that are available in patients with SMM—specifically those with ≥ 10% plasma cells on bone marrow biopsy at SMM diagnosis—and serial measurements of monoclonal protein, light chains, and other routinely measured parameters. The training cohort included 1,031 patients with SMM (with 231 progressing to MM). The four validation cohorts included a total of 1,239 patients with SMM (with 385 progressing to MM). The median number of timepoints (visits with laboratory measurements) was 4 in the training cohort (interquartile range 2–8), over a median follow-up time of 3.7 years (interquartile range 1.8–7.1). In validation cohorts, the median number of timepoints was between 3 and 7, over a median follow-up time of 1.6 to 5.3 years. The distribution of disease and demographic characteristics and follow-up data is detailed in Table 1, with the distribution of biomarkers at baseline available in **Supplementary Table S1**. Unstratified time-to-progression was comparable across cohorts, with 2-year progression rates of 12.1% in the training cohort and 12.0% in validation cohort 1 and broadly similar results in the other cohorts (**Supplementary Figure S1**). In the large cohorts (training cohort and validation cohorts 1 and 2), unstratified yearly progression rates were ~ 5% per year for the first 5 years and ~ 3% per year afterwards (**Supplementary Figure S1**).

From a baseline multivariate Cox model trained to predict progression to MM using established biomarkers (latest values of M-protein, involved/uninvolved sFLC ratio, creatinine, and BMPC) in the training cohort, we used cross validation to identify the independent added value of adding time-varying biomarker trajectories to the model. These trajectories were used to provide information beyond merely the current static biomarker values. We identified four trajectory variables—evolving M-protein, creatinine, FLC ratio, and hemoglobin—that significantly improved risk predictions compared to using only static biomarkers. The trajectory variables with associated hazard ratios are defined in Table 2. Trajectory changes were detected in 2.9–21.2% of the total visits (and 9.3–46.1% of patients) in the training cohort, depending on the dynamic biomarker considered (e.g., hemoglobin vs. M-protein trajectory; **Supplementary Table S2**). Evolving trajectories were detected most frequently for M-protein and least frequently for sFLC ratio. Optimal trajectory definitions were very similar when removing BMPC from the Cox models (in terms of optimal trajectory variables and cross-validated C-statistics), and therefore we used the same trajectory definitions in our updated PANGEA 2.0 no BM model to avoid conflicting trajectory definitions and keep the detection of dynamic changes consistent across models.

Based on those definitions, we trained the PANGEA 2.0 BM and no BM models which predict MM progression risk using both current biomarkers and trajectory biomarkers. All of the trajectory biomarkers were significant (at a 5% false discovery rate using the Benjamini-Hochberg procedure), indicating additional levels of progression risk beyond current biomarker levels (**Supplementary Table S3**). We developed an open-access, online calculator that allows users to enter patient data and view the resulting risk predictions for the PANGEA 2.0 BM or no BM model (https://ghobrial.shinyapps.io/pangea_2_calculator).

**Figure 1:**
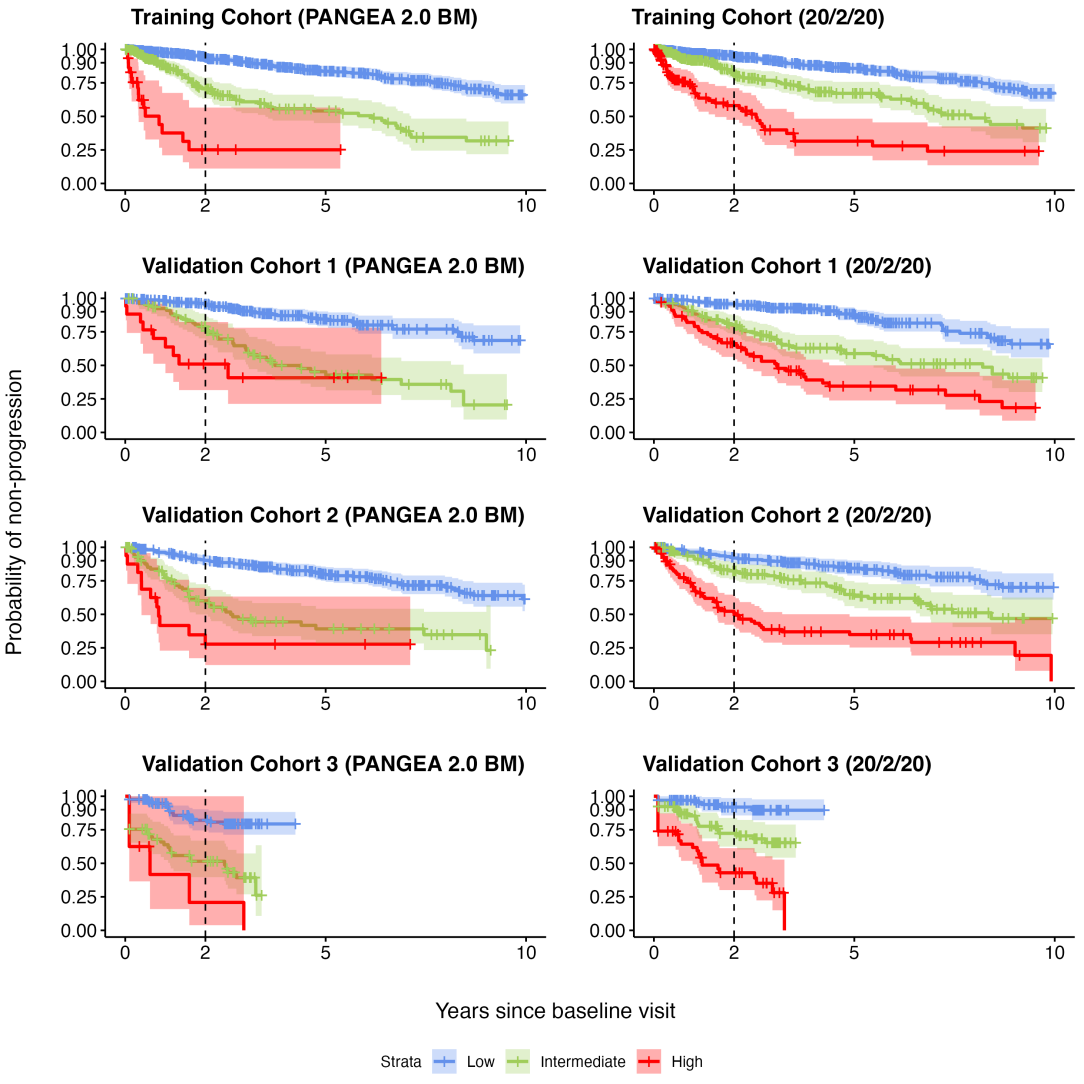
Risk stratification of the PANGEA trajectory model (BM) compared to rolling 20/2/20 for the training cohort and validation cohorts 1-3. Plots show Kaplan-Meier estimates non-progression probability stratified by risk category (PANGEA or 20/2/20). Error bands show pointwise 95% confidence intervals for the probability of non-progression by each time point.

The PANGEA 2.0 trajectory risk predictions—when stratified into high-, intermediate-, and low-risk groups—led to improved risk classification compared to 20/2/20. Patients were classified as high-risk if their predicted 2-year risk of progression to MM was greater than 40%, intermediate-risk if their predicted 2-year risk was between 10% and 40%, and low-risk if their predicted 2-year risk was less than 10%. Importantly, the actual progression rates of the so-called high-risk patients were higher for the PANGEA 2.0 classification than for the 20/2/20 classification (Fig. 1, **Supplementary Figure S3**, Table 3). For example, in validation cohort 1 the PANGEA 2.0 BM high-risk group had a 2-year progression rate of 49.0% (95% CI: 17.7–68.4%) while the 20/2/20 high-risk group had a rate of only 33.2% (95% CI: 20.8–43.7%). Similar differences were observed in the other validation cohorts (Table 3). PANGEA 2.0 BM high-risk status also had higher positive predictive values than 20/2/20 high-risk status (e.g., 73.9% vs. 44.5% at 2 years in validation cohort 1), and both had similar negative predictive values (e.g., 87.6% vs. 88.6% at 2 years in validation cohort 1). Full results on the predictive value of PANGEA 2.0 and 20/2/20 high-risk status are described in **Supplementary Figures S4 and S5**.

In comparison, we examined both 20-2-20 and IMWG models in our cohorts, time-to-progression stratified by baseline 20/2/20 risk category was similar across cohorts, with 2-year progression rates similar to the original Mayo results^3^ of 6.2% for the low-risk group, 17.9% for the intermediate-risk group, and 44.2% for the high-risk group (Fig. 1). Time-to-progression stratified by the IMWG 4-factor risk category including cytogenetics was also consistent with previously published results^3^ (**Supplementary Figure S2**).

**Figure 2:**
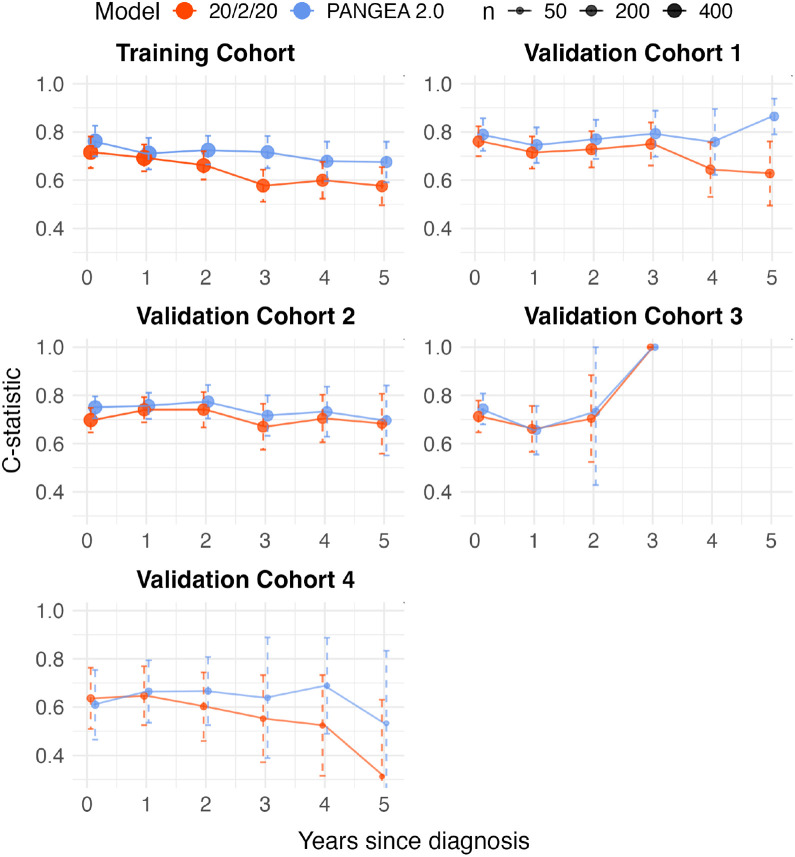
Point estimates and 95% confidence intervals of dynamic C-statistics for 20/2/20 and the PANGEA 2.0 BM model for risk prediction in the validation cohorts. The C-statistic at each time point (x-axis) is computed using only the most recent observation for each remaining patient who is still being followed up that many years after diagnosis.

When looking at individualized predictions, the PANGEA 2.0 trajectory models improved the ranking accuracy of risk predictions over the rolling 20/2/20 model (i.e., 20/2/20 computed using the patient’s latest biomarker measurements^22^) as measured by increases in time-specific and overall C-statistics (Fig. 2, **Supplementary Table S4**). The C-statistic measures the ability of prediction models to correctly rank patients by how quickly they indeed progress (i.e., the degree to which patients with higher risk scores also have shorter times to progression). For example, in validation cohort 1 the PANGEA trajectory BM model had an overall C-statistic relative increase of 10% over 20/2/20 (from 0.76 [95% CI: 0.72–0.80] to 0.84 [95% CI: 0.79–0.88]) and 5-year C-statistic relative increase of 37% (from 0.63 [0.50–0.76] to 0.86 [0.79–0.94]). Comparable results were seen for the other validation cohorts (**Supplementary Table S4**). In cohorts with fewer serial observations per patient and few cases of increasing biomarker trajectories, the PANGEA 2.0 trajectory models had very similar C-statistics to the PANGEA 2.0 models without trajectories, and both typically showed improved C-statistics over the rolling 20/2/20 model (**Supplementary Table S4**).

**Figure 3:**
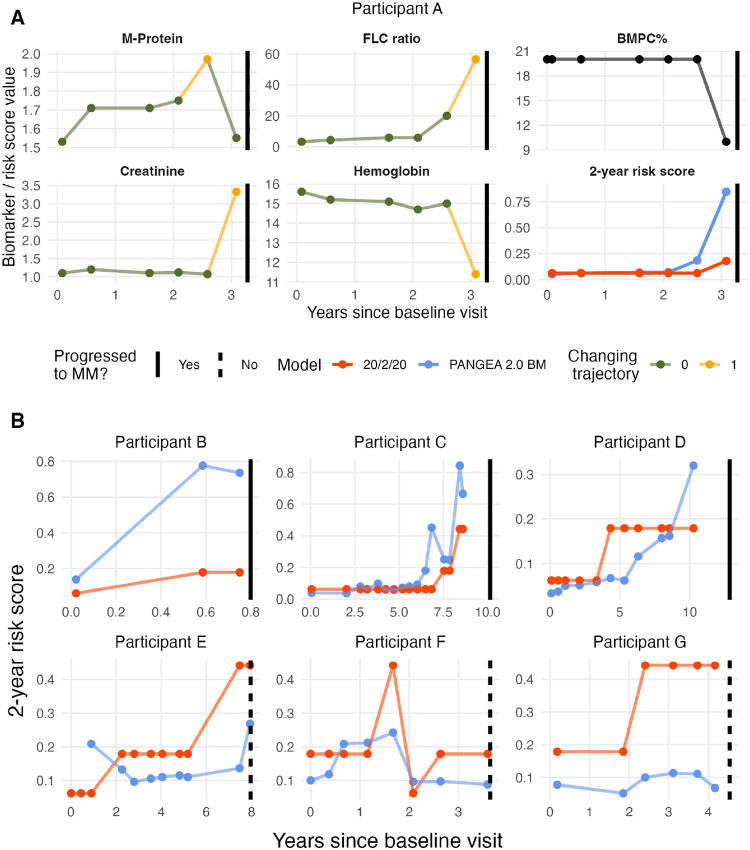
A) Example biomarker trajectories and 2-year progression risk scores (PANGEA 2.0 with BM and rolling 20/2/20) for a patient. The line color for M-protein, FLC ratio, creatinine, and hemoglobin indicates whether a changing trajectory is detected at each timepoint. Rolling 20/2/20 risk increases at the latest time point, where the patient already shows signs of progression to myeloma (anemia and acute kidney failure). In contrast, a skewed M-protein concentration is observed at the penultimate lab report, which translates through PANGEA 2.0 as an increased predicted risk of progression. B) Predicted risk scores for (first row) three progressor patients with higher PANGEA 2.0 risk and lower 20/2/20 risk and for (second row) three non-progressor patients with lower PANGEA 2.0 risk and higher 20/2/20 risk.

To illustrate the new model, we depicted the biomarker trajectories, PANGEA 2.0 2-year risk predictions, and 20/2/20 2-year risk prediction for an example patient (Fig. 3A). This patient never had an M-protein concentration above 2 g/dL and was never considered high-risk by the 20/2/20 model. However, their increasing trends in creatinine and FLC ratio after 2.5 years led the PANGEA 2.0 trajectory models to detect a very high risk of progression. They progressed to MM 8 months after the detection of skewed M-protein trajectory by the PANGEA 2.0 model (predicted risk increasing from 7.1–18.4%), 6 months earlier than 20/2/20 detected a similar level of risk. Figure 3B shows the predicted risk scores for (first row) three progressor patients with higher PANGEA 2.0 risk and lower 20/2/20 risk and for (second row) three non-progressor patients with lower PANGEA 2.0 risk and higher 20/2/20 risk. These cases were selected to illustrate that there can be substantial differences between the risk predictions of PANGEA 2.0 and 20/2/20.

To confirm the calibration of the PANGEA 2.0 trajectory model, we compared its average prediction of the 2-year progression risk to the actual progression rates. In validation cohort 1, the PANGEA 2.0 BM model consistently gave risk predictions within 3% of the actual rate of progression for subgroups defined by 20/2/20 category, M-protein concentration, sFLC ratio, BMPC, creatinine, and age (**Supplementary Table S5**). The no BM model showed similar calibration except when stratified by BM or high sFLC ratio. This level of accuracy is comparable to or better than the rolling 20/2/20 model, and similar results were observed the other cohorts (**Supplementary Tables S6-S8**), except for validation cohort 3 where PANGEA 2.0 no BM showed better calibration results than PANGEA 2.0 BM.

FISH cytogenetic markers improve risk predictions, for example in the IMWG model^3^. In our training cohort, cytogenetic markers, including t(4;14), 17p deletion, and 1q gain, had significant independent associations with MM progression in univariate analysis (**Supplementary Table S9**). We observed evidence that translocations t(14;16) and t(14;20) were significant when added to the PANGEA 2.0 BM model, but the other translocations (including MYC rearrangements) and hyperdiploidy alone were not (**Supplementary Table S9**). We then tested the combined variable integrating multiple cytogenetic abnormalities defined by the International Myeloma Society in 2024^23^: as any of t(4;14), t(14;16), or t(14;20) combined with either 1q gain or 1p deletion. We found it was associated with a significantly increased risk of progression when added to the PANGEA 2.0 model, further improving on the PANGEA model with the new IMS/IMWG cytogenetic risk stratification. Interestingly, the combination of markers used in the IMWG model of SMM^3^ (any of hyperdiploidy, 13q deletion, 1q gain, or translocation t(4; 14)) was not significant when added to the PANGEA 2.0 model but did have a significant independent association with MM progression. In the training cohort, cytogenetic abnormalities (of any kind) were observed in 47.2% of PANGEA 2.0 BM high-risk cases, 41.2% of PANGEA 2.0 BM intermediate-risk cases, and 33.1% of PANGEA 2.0 BM low-risk cases. Similarly mixed results were seen in validation cohort 2 (**Supplementary Table S10**).

Finally, in order to facilitate scientific discussion and transparency, we created an open-access validation application (https://ghobrial.shinyapps.io/pangea_validation/). In the application, users can explore the performance (ranking accuracy and calibration) of PANGEA 2.0 and 20/2/20 in flexible patient subpopulations defined by demographics and biomarkers (e.g., age, sex, and M-protein). This enables the investigation of many more subpopulations than can be discussed in standard tables and figures and can help identify areas in which current risk prediction models have greater room for improvement.

## Discussion

In this study, we assembled a cohort of 2,270 patients with SMM with longitudinal clinical and biological follow-up data. We used time-varying multivariate Cox regression models to leverage the information brought by the fine variation of biomarkers for detecting early progression patterns and improve on the current criteria already included in the 20/2/20 and IMWG models. Through global collaborations across six international centers, we were able to define new evolving criteria of the PANGEA 2.0 stratification model in patients with SMM.

The diagnosis of SMM defines a subgroup of patients with precursor conditions at a higher, yet very heterogenous, risk of progression to MM. Current static criteria and risk models are limited to guiding clinical management and early intervention. The plurality of risk stratification models and the diversity of biomarkers highlighted with a significant added value for predicting progression from SMM to MM may lead to confusion and limit the interpretation and applicability of clinical trials. Multiple clinical trials use different criteria, models, and risk groups that developed over the recent years^24-26^. A homogenous and simple approach is critical for consistency in clinical routine, but it must also be accurate. The 20/2/20 model developed by Lakshman et al. in 2018, which used BMPC, sFLC ratio, and serum M protein levels, is simple to stratify patients^27^ but lacks accuracy in predicting dynamic changes in progression and cannot be used at the individual level of patients. Additionally, the IMWG score by Mateos *et al.* in 2020 requires cytogenetic data, which may not be available in a significant number of SMM patients due to the inability to enrich enough BMPCs for FISH analyses. A unified, straightforward, and precise risk stratification model incorporating dynamic biomarkers is essential to facilitate the implementation of therapeutic strategies and improve patient outcomes in SMM.

In recent years, multiple studies have demonstrated an interest in analyzing evolving trajectories of biomarkers and their inclusion in risk stratification models^4-12^. Trajectory definitions were proposed for hemoglobin (increasing anemia) in the initial PANGEA model^28^, followed by evolving M-protein and/or total immunoglobulin increase, and evolving sFLC, and were associated in multiple studies with a significantly shorter time-to-progression to MM. However, most early approaches were limited to comparing follow-up values to the baseline value, limiting the interception of rapidly evolving patterns late after diagnosis or re-assessment of evolving patterns after this period. Furthermore, even more recent attempts were made on limited numbers of patients, leading to varying definitions and thresholds for considering each biomarker evolving^6,10^.

In our study, we confirmed the interest in observing biomarker trajectories, and found four evolving definitions that are significantly associated with shorter time-to-progression and that can be used in the clinic with data already routinely collected for patients followed for precursor conditions: an M-protein increase ≥ 0.2 g/dL within 18 months; an FLC ratio increase ≥ 20 within 24 months; a creatinine increase ≥ 25% within 12 months; and a hemoglobin decrease ≥ 1.5 g/dL within 12 months. Those four definitions can be reassessed at any time during follow-up, regardless of baseline values or time of follow-up of the patient. Cox models demonstrated that each of those four evolving definitions independently improves risk stratification. Based on this approach, we identified clear definitions that are easy to understand and transparently demonstrate how dynamic variations impact the results of the PANGEA 2.0 models. This also allows for incorporating these evolving definitions into two stratification models, enabling prediction based on the availability of bone marrow results in addition to peripheral blood biomarkers.

The PANGEA 2.0 model is adaptable whether there are preceding markers available and can be used even in the absence of a recent BM biopsy or FISH results, making it an easy model to use for patients worldwide. PANGEA 2.0 performs accurately at baseline, or when no preceding values are available. In addition, we developed two distinct models which allow risk stratification even when bone marrow results are unavailable or not available at the same time points as compared to blood biomarkers.

PANGEA 2.0 can also classify patients into groups of low, intermediate, or high risk of progression to MM. This information could be used at baseline or any time during follow-up to guide inclusion in clinical trials. Furthermore, our results confirm that patients classified as high-risk by PANGEA 2.0 have a higher actual progression rate and positive predictive value at 2 years compared to patients classified as high-risk by 20/2/20, of 15.8 to 33.0%. This demonstrates the more rigorous prediction of PANGEA 2.0 to detect patients who are truly high risk SMM and may potentially benefit from early therapy.

Several metrics showed the robustness of our evolving definitions and fitting of the PANGEA 2.0 models. While various unrelated conditions can impact static values of hemoglobin and creatinine, our evolving definitions of creatinine and hemoglobin were detected in < 5% of visits (< 20% of patients) in our training cohort, suggesting a higher specificity for SMM progression of their dynamic evolution compared to assessment of their static value.

Interestingly, adding FISH results to the PANGEA 2.0 BM model improved stratification. In our results, t(14;20) alone and the combined variable of the new IMS/IMWG criteria defined by one cytogenetic abnormality among t(4;14), t(14;16), and t(14;20) combined with either 1q gain or 1p deletion were associated with a significantly increased risk of 2-year progression compared to the prediction of the PANGEA 2.0 BM model. This may be due to certain FISH abnormalities influencing biomarker dynamics that PANGEA 2.0 can effectively capture, while others eventually lead to progression independently of short-term dynamics. By integrating FISH using optimal probe combinations and leveraging PANGEA 2.0’s ability to model disease trajectories, risk stratification in SMM patients is notably improved.

Although this model performs better than the current 20/2/20 model and uses the new cytogenetic classification, further refinement of the model could be achieved by leveraging next-generation sequencing from blood and BM samples as well as the addition of circulating tumor cells, and the use of highly sensitive technologies for detecting M-protein levels, such as mass spectrometry, which can also be included in future studies^16,29-30^. It should also be noted that despite the number of centers included in our study, racial and ethnic diversity was limited or not documented in our validation cohorts, given that most of those validation studies were conducted in Europe where race is not captured. Further studies will be needed to allow for the generalization of risk progression assessment in diverse populations and define an optimal observation frequency to improve risk prediction.

In conclusion, we developed PANGEA 2.0 using one of the largest cohorts identified to date, with 2,270 SMM cases and international validation cohorts from five distinct institutions. This international collaborative effort demonstrated that PANGEA 2.0 outperforms current criteria for SMM stratification, including 20/2/20 and IMWG models. PANGEA 2.0 is accessible online as an easy-to-use tool that can be immediately integrated into clinical practice. While PANGEA 2.0 can be used for baseline stratification, it can be used as well in serial follow-up and leverage the analysis of evolving biomarker trajectories to detect early patterns of progression, outperforming current criteria, regardless of the granularity of the follow-up and number of visits per year. The addition of bone marrow and FISH variables are also critical but not necessary for the use of the PANGEA model, making it easy and available to use in all clinical practices.

PANGEA 2.0 models are available as a free open-access web application at https://ghobrial.shinyapps.io/pangea_2_calculator/, and user-driven validation analyses can be conducted at https://ghobrial.shinyapps.io/pangea_validation/.

## Methods

### Cohorts and patients

The PANGEA (Precursor Asymptomatic Neoplasms by Group Effort Analysis) project is based on a cohort of patients with precursor conditions for MM identified at Dana-Farber Cancer Institute (DFCI, Boston, MA, USA) for which longitudinal follow-up data including clinical and biological variables was collected and curated between 3/25/2021 and 10/21/2024. Among this cohort, 1,031 patients diagnosed with SMM were included as a training cohort in this study. Model validation is based on four independent cohorts of patients with SMM from five international centers. Cohort 1 included 380 and 105 cases from the National and Kapodistrian University of Athens (Athens, Greece) and the University College London (UCL; London, UK); Cohort 2 included 447 cases from the Heidelberg University Hospital (UKHD, Heidelberg, Germany); Cohort 3 included 240 cases from the University of Navarra (Pamplona, Spain); and Cohort 4 included 67 cases from the University of Milan (Milan, Italy). This study was approved by the DFCI Institutional Review Board (#21-127) in accordance with the Declaration of Helsinki. In accordance with ethical guidelines, our study was granted a waiver of informed consent by the institutional review board due to its retrospective design.

### Clinical Annotation

For the training cohort, we collected baseline characteristics of patients at the date of diagnosis of SMM, including age, race, ethnicity, sex, height, and immunofixation isotype. We collected follow-up data with a median of two visits per year starting from the date of diagnosis of SMM until the date any of the following events occurred first: progression to active MM defined by SLIM-CRAB criteria, last follow-up visit, start of precursor treatment, or death. Follow-up data included patient information relevant for the diagnosis and follow-up of MM and precursor conditions, including the following blood/serum values: total protein, IgA, IgM, IgG, kappa and lambda free light chains (FLC), sFLC ratio, calcium, creatinine, albumin, hemoglobin, LDH, beta-2 microglobulin, M-protein(s) concentration. Other collected variables include imaging, weight, and therapy (including bisphosphonate use). Data from all bone marrow (BM) biopsies annotated for patients during this follow-up and extracted BMPC and fluorescence *in-situ* hybridization (FISH) findings, when available, were collected. FISH data was structured into one of four categories: positive, negative, not tested, or unavailable. The following aberrations were captured: translocations t(4;14), t(6;14), t(11;14), t(14;16), t(14;20), t(14;18), −17/17p deletion, 6q deletion, 11q22 deletion, 1q gain, 8q24/MYC rearrangements, −13/13q deletion, +3/+7 hyperdiploid, +9/+15 hyperdiploid, trisomy 4, trisomy 12, and trisomy 18.

For the validation cohorts, we extracted the targeted outcomes, time to progression, censoring, or death, and the biological data required by PANGEA 2.0 analysis at initial and follow-up visits.

### Defining trajectories and evolving biomarkers

For each of the four biomarkers in Table 2 we defined binary (0/1) trajectory variables indicating if the biomarker has increased/decreased in a way that markedly elevates the risk of progression to MM, *beyond simply knowing the current biomarker value*. We considered seven different candidate definitions of these binary trajectory variables, and various thresholds. Candidate definitions were:

1. the biomarker has increased by at least X% compared to any of the previous values in the past Y months;
2. the biomarker has increased by at least X [absolute increase] compared to any previous value in the past Y months;
3. the biomarker has increased by at least X% compared to the previous value;
4. the biomarker has increased by at least X [absolute increase] compared to the previous value;
5. the biomarker has increased by at least X [absolute increase] compared to the previous value and is at least as high as 90% of the maximum of all previous values;
6. the average change (slope, based on ordinary least squares (OLS) regression) of the biomarker over the past Y months is greater than X;
7. the average change (slope) of the biomarker over the last K observations is greater than X.

The complete list of candidate thresholds (with X, Y, and K values) tested can be found in **Supplementary Table S11**. For the hemoglobin trajectory, we considered decreases (not increases) in definitions 1-7.

To determine the definition of each biomarker’s trajectory feature, we used a systematic grid search to evaluate the improvement from adding each candidate binary feature to a “basic” model including only current biomarkers.

The baseline model was a multivariate Cox regression with time-varying biomarkers trained only with four biomarkers (i.e., latest values of M-protein, involved/uninvolved sFLC ratio, creatinine, BMPC). Then, each candidate time-varying trajectory indicator variable was added one at a time as a predictor in the baseline model, and we computed the model’s C-statistic by 5-fold cross-validation (using only the training dataset). The optimal candidate trajectory definition for each biomarker was the definition which gave the greatest increase in C-statistic over the baseline model.

### Training PANGEA 2.0 models

The PANGEA 2.0 models are two multivariate Cox regression models with time-varying predictors, namely the “BM” and “no BM” models. Both include effects for three biomarkers (M-protein, log involved/uninvolved FLC ratio, log creatinine) and age, as well as M-protein trajectory, involved/uninvolved sFLC ratio trajectory, creatinine trajectory, and hemoglobin trajectory. The "BM" model also includes BMPC as a predictor, while the “no BM” model does not and can be used when recent BMPC is not available. The trajectory variables are defined to be 0 (not missing) when patient history is not available, so the models can be used without patient history. The models were estimated using the survival^31^ package (version 3.7-0) in R^32^ (version 4.4.2), and output risk scores defined as the probability of progressing to MM within two years of the latest visit. Death was not treated as a competing risk, due to rare frequency in our training and validation cohorts (<10%)^33^.

We also assessed whether cytogenetic markers measured by FISH improve the predictions of the PANGEA 2.0 BM model. Due to sample size limitations, we analyzed each FISH probe separately, adding it as a single new predictor in the PANGEA 2.0 BM Cox model.

### Validating PANGEA 2.0 models

We evaluated the ranking accuracy, risk stratification, and calibration of the PANGEA 2.0 models and rolling 20/2/20 on each validation cohort. “Rolling 20/2/20” refers to the low-intermediate-high risk categories based on Lakshman *et al.*, 2018^2^, computed using the patient’s latest biomarker measurements^22^. We also evaluated versions of the two PANGEA 2.0 models that based risk predictions only on the latest biomarker information (i.e., all trajectory variables set to 0), in order to assess predictive performance when patient history is not available. Overall ranking accuracy was assessed for each model by generalized C-statistics^34^ including all serial observations for each patient. Dynamic ranking accuracy for each model was assessed by computing C-statistics based only on each patient’s most recent visit at 0.1, 1, 2, 3, 4, or 5 years after baseline.

Although PANGEA 2.0 produces personalized, continuous 2-year risk scores (i.e., progression probabilities between 0% and 100%), we also assessed its ability to stratify patients into low, intermediate, and high-risk groups. This stratification was based on PANGEA 2.0’s predicted risk of progression within 2 years, with “low” risk patients having less than 10% predicted risk, “intermediate” risk patients having between 10% and 40% predicted risk, and “high” risk patients having greater than 40% predicted risk. We then computed Kaplan-Meier progression curves stratified by these risk groups and compared results to 20/2/20. We also evaluated the dynamic accuracy of PANGEA 2.0 and 20/2/20 high-risk status as predictors of progression to MM within 2 years. This was done using standard inverse probability of censoring estimates of the time-dependent positive and negative predictive values^34-36^ based on each patient’s most recent visit at 0.1, 1, 2, 3, 4, or 5 years after baseline.

Finally, we assessed calibration of the PANGEA 2.0 models, which refers to the level of agreement between the predicted and observed progression rates. For each entire validation cohort and various subcohorts (stratified by low vs. high biomarkers), we computed (a) the average 2-year PANGEA risk of progression and (b) the actual 2-year rate of progression (based on Kaplan-Meier analysis). We compared these progression rates to 20/2/20 (based on the reported 2-year progression rates in Mateos *et al.*, 2020^3^).

### Open science validation application

We developed an open-access web application to evaluate the performance of PANGEA 2.0 and alternative models on our training data as well as a subset of the validation cohorts. Using the application, users can specify a dataset and subpopulation of interest (e.g., female patients at DFCI over age 60 with FLC ratio > 20) and see the ranking accuracy and calibration of PANGEA 2.0 compared to 20/2/20. This application allows users to compare the performance of PANGEA 2.0 (BM and no BM models) with 20/2/20 in flexible and detailed populations, facilitating both decision-making about appropriate populations to use each model and future research on risk models for MM.

### Clinical Calculator

To easily use PANGEA 2.0 in the clinic, we developed an open-access web application. The application allows entering the individual’s values (M-protein concentration, FLC ratio, creatinine, hemoglobin, ± BMPC) along with dates of measurement. Based on the filled in information, PANGEA 2.0 automatically calculates the trajectories of biomarkers when past values are available and identifies the relevant model for analyzing the patient’s data (no-BM, BM). Accordingly, PANGEA 2.0 determines the personalized risk of progression to MM for the patient, and classifies them into groups of low, intermediate, or high risk of progression to MM by comparing their personalized risk to the thresholds described above.

## Figures and Tables

**Table 1:** Patient demographics of training and validation cohorts of the PANGEA project to integrate biomarker trajectories into progression risk prediction for smoldering multiple myeloma patients. *IQR: interquartile range*.

	Training (n = 1031)	Validation 1 (n = 485)	Validation 2 (n = 447)	Validation 3 (n = 240)	Validation 4 (n = 67)
Age at diagnosis (years) – median (IQR)	61.7 (53.3–69.0)	63.8 (54.0–72.9)	60.0 (52.0–67.0)	66.0 (58.0–74.3)	67.7 (58.5–76.6)
Clinical laboratory visits – median (IQR)	4 (2–8)	5 (3–8)	5 (2–10)	3 (1–5)	7 (4–11)
Follow up (years) – median (IQR)	3.7 (1.8–7.1)	5.3 (3.0–9.7)	3.4 (1.4–6.3)	1.6 (0.7–2.7)	3.4 (2.3–6.4)
Time b/w visits (months) – median (IQR)	6.0 (3.7–7.6)	5.5 (3.4–8.5)	3.9 (3.0–6.2)	6.0 (4.0–6.7)	4.1 (3.4–4.9)
Sex – n (%)					
Female	504 (49%)	278 (58%)	196 (44%)	122 (51%)	30 (45%)
Male	527 (51%)	206 (42%)	251 (56%)	118 (49%)	37 (55%)
Race – n (%)					
Asian	26 (3%)	17 (4%)			
Black	86 (8%)	19 (4%)			1 (1%)
White	882 (86%)	429 (88%)			66 (99%)
NA	37 (4%)	19 (4%)	447 (100%)	240 (100%)	
MM progressor – n (%)					
No	800 (78%)	326 (67%)	307 (69%)	181 (75%)	40 (60%)
Yes	231 (22%)	159 (33%)	140 (31%)	59 (25%)	27 (40%)
Immunofixation – n (%)					
Biclonal	7 (1%)	7 (1%)	8 (2%)	48 (20%)	1 (1%)
IgA	236 (6%)	123 (25%)	114 (26%)	63 (26%)	16 (24%)
IgG	723 (70%)	338 (70%)	303 (68%)	123 (51%)	48 (72%)
Light Chain Only	62 (6%)	12 (2%)	16 (4%)		1 (1%)
NA	10 (1%)	5 (1%)	1 (0%)	6 (3%)	
Censored due to – n (%)					
Death	22 (2%)	12 (2%)	16 (4%)	12 (5%)	
Early intervention	233 (23%)	5 (1%)	16 (4%)		

**Table 2:** Definitions of the biomarker trajectory variables in the PANGEA 2.0 models (BM and no BM), as well as the hazard ratio for the PANGEA 2.0 BM model in the training cohort.

Trajectory	Definition	Hazard Ratio (95% CI)
M-Protein	current M-protein concentration has increased by at least 0.2 g/dL compared to any value in the previous 18 months	1.72 (1.20–2.47)
sFLC Ratio	current sFLC ratio has increased by at least 20 compared to any value in the previous 24 months	2.02 (1.23–3.31)
Creatinine	current creatinine has an increase of at least 25% over any values in the previous 12 months	1.94 (1.13–3.32)
Hemoglobin	current hemoglobin has decreased by at least 1.5 g/dl compared to any value in the previous 12 months	3.21 (1.98–5.22)

**Table 3:** 2-year progression rates (95% CI) by PANGEA 2.0 (BM) and 20/2/20 risk categories, in percentages, based on extended Kaplan-Meier estimates including all patient visits. *N/A: progression rate and CI could not be calculated due to small sample sizes*.

	Training Cohort	Validation Cohort 1	Validation Cohort 2	Validation Cohort 3	Validation Cohort 4
Low Risk 20/2/20	5.1 (3.1–7.1)	4.2 (1.6–6.7)	8.0 (4.1–11.9)	8.1 (1.4–14.3)	9.4 (0–21)
PANGEA 2.0	6.1 (3.9–8.3)	4.3 (1.6–6.8)	9.7 (6–13.3)	18 (9.7–25.5)	9.4 (0–19.1)
Intermediate Risk 20/2/20	18.6 (12.6–24.2)	20.2 (12.8–27)	17.6 (10.8–23.9)	27.7 (16.2–37.5)	4.8 (0–13.4)
PANGEA 2.0	29.4 (21.0–36.9)	21.2 (11.4–29.9)	39.8 (27.6–50.0)	48.5 (33.1–60.3)	26.2 (3.3–43.6)
High Risk 20/2/20	41.9 (28.9–52.5)	33.2 (20.8–43.7)	49.2 (36.7–59.2)	57.1 (38.7–69.9)	33.3 (3.9–53.7)
PANGEA 2.0	74.9 (43.4–88.9)	49.0 (17.7–68.4)	72.2 (36.7–87.8)	79.2 (0–96.1)	N/A

## Data Availability

Aggregate and deidentified data can be shared to investigators following appropriate proposal to the corresponding author (irene_ghobrial@dfci.harvard.edu) and after approval by the study team. Data from validation cohorts can only be shared by request at the respective institutions.

## Code availability

Code to compute risk predictions for the PANGEA 2.0 models (BM and no BM) on new data, and used to compute the validation analyses is available at https://github.com/pangea-study/pangea_2.0.
